# Efficient antitumor effect of co-drug-loaded nanoparticles with gelatin hydrogel by local implantation

**DOI:** 10.1038/srep26546

**Published:** 2016-05-26

**Authors:** Hao Zhang, Yong Tian, Zhenshu Zhu, Huae Xu, Xiaolin Li, Donghui Zheng, Weihao Sun

**Affiliations:** 1 Department of Geriatrics, the First Affiliated Hospital to Nanjing Medical University, Nanjing, 210029, China; 2 Department of Orthopaedics, Jiangsu Taizhou People’s Hospital, Taizhou, 225300, China; 3 Key Laboratory of Drug Quality Control and Pharmacovigilance (China Pharmaceutical University), Ministry of Education, Department of Pharmaceutical Analysis, China Pharmaceutical University, Nanjing 210009, China; 4 Department of Chemical and Biomolecular Engineering, National University of Singapore, Singapore, 117585; 5 Department of Pharmacy, the First Affiliated Hospital to Nanjing Medical University, Nanjing, 210029, China; 6 Department of Nephrology, Huai'an Hospital Affiliated with Xuzhou Medical College and Huai'an Second Hospital, Huai'an 223002, China

## Abstract

Tetrandrine (Tet) could enhance the antitumor effect of Paclitaxel (Ptx) by increasing intracellular Reactive Oxygen Species (ROS) levels, which leads to the possibility of co-delivery of both drugs for synergistic antitumor effect. In the current study, we reported an efficient, local therapeutic strategy employing effective Tet and Ptx delivery with a nanoparticle-loaded gelatin system. Tet- and Ptx co-loaded mPEG-PCL nanoparticles (P/T-NPs) were encapsulated into the physically cross-linked gelatin hydrogel and then implanted on the tumor site for continuous drug release. The drug-loaded gelatin hydrogel underwent a phase change when the temperature slowly increased. *In vitro* study showed that Tet/Ptx-loaded PEG-b-PCL nanoparticles encapsulated within a gelatin hydrogel (P/T-NPs-Gelatin) inhibited the growth and invasive ability of BGC-823 cells more effectively than the combination of free drugs or P/T-NPs. *In vivo* study validated the therapeutic potential of P/T-NPs-Gelatin. P/T-NPs-Gelatin significantly inhibited the activation of p-Akt and the downstream anti-apoptotic Bcl-2 protein and also inducing the activation of pro-apoptotic Bax protein. Moreover, the molecular-modulating effect of P/T-NPs-Gelatin on related proteins varied slightly under the influence of NAC, which was supported by the observations of the tumor volumes and weights. Based on these findings, local implantation of P/T-NPs-Gelatin may be a promising therapeutic strategy for the treatment of gastric cancer.

Paclitaxel (Ptx) has been recommended by the National Comprehensive Cancer Network (NCCN) as one of the main components of a first-line chemotherapeutic regimen in the treatment of advanced gastric cancer^1^,^2^. However, the therapeutic efficacy of Ptx is attenuated by the following two restrictions. The first hindrance lies in the emerging resistance of cancer cells to intracellular oxidative levels^3^. Second, as reported in earlier studies, while cancer cells are characterized by an intrinsically high anti-oxidative defense system, additional accumulation of intracellular reactive oxygen species (ROS) could induce apoptosis^4^. Moreover, it was previously reported that the antitumor effect of Ptx depended mainly on the redox levels in tumor cells, with the generation of hydrogen peroxide being an early and critical step for its cytotoxicity^5^,^6^. Therefore, total cellular antioxidant capacity is a critical determinant of cellular sensitivity to Ptx. It is rational to propose the strategy of "oxidation therapy" to conquer cellular antioxidant capacity, by co-administering pro-oxidants to enhance the efficacy of Ptx. In previous reports by our group and others, Tetrandrine (Tet), a type of herbal medicine, has been proved to be effective in enhancing intracellular ROS levels, thereby leading to the apoptosis of cancer cells^7^,^8^,^9^. We have demonstrated that Tet could enhance the cytotoxicity of Ptx through activation of ROS-dependent apoptotic pathways. As a result, co-delivery of Tet and Ptx may be a promising approach to overcome the resistance against Ptx in cancer cells^10^.

Another limitation for the therapeutic efficacy of Ptx is its severe side effects, which are caused by the nonspecific distribution of special solvents, Cremophor EL^11^. A potential way to overcome the nonspecific distribution is by utilizing polymeric nanoparticles to deliver Ptx, which can accumulate at the tumor site through the Enhanced Permission and Retention effect (EPR effect)^12^,^13^,^14^,^15^. In our previous *in vitro* study, we demonstrated that co-delivery of Tet and Ptx in mPEG-PCL nanoparticles resulted in synergistic cytotoxicity through ROS-dependent Akt and apoptotic pathways^10^. *In vivo* evaluation, via intratumoral delivery, also supported the superior antitumor efficiency of Ptx and Tet co-loaded nanoparticles against liver cancer^16^.

However, limitations to achieving the optimal effect for intratumoral administration still exist. It is difficult for the drug-loaded nanoparticles to penetrate deep into the tumor mass and exert their growth inhibitory effects on cancer cells that are distant from the injection site^17^,^18^. Therefore, it is noted that cellular necrosis is observed at the injection site, while the whole tumor mass grows as before. Our previous studies have demonstrated that peritumoral administration is superior to intratumoral injection, in that the diffusion of the loaded drug throughout the tumors is greatly improved, resulting in enhanced inhibition of tumor growth. Peritumoral administration is characterized by prolonged tumor exposure and reduced systemic toxicity^19^.

Based on the previous findings, we developed a co-delivery system of Tet and Ptx in order to achieve optimal effects. First, Tet and Ptx were co-encapsulated into PEG-b-PCL nanoparticles. Then, Tet and Ptx co-loaded PEG-b-PCL nanoparticles were encapsulated into a physically cross-linked gelatin hydrogel. This type of drug delivery system (P/T-NPs-Gelatin) may accomplish our goal based on the following rationale (Fig. 1). First, gelatin hydrogel has a phase-shift from solid to liquid as the temperature increases. When delivered into the body, the body temperature will slowly liquefy it, resulting in continuous release of the encapsulated nanoparticles over a relatively long time scale. Second, directly implanting the gel onto the tumor will greatly increase the contact area between the gel and the tumor, thereby accelerating the diffusion and penetration of the drug-loaded nanoparticles inside the tumor through tumor vessels. In the current work, a series of physiochemical and biological evaluations were performed to characterize this novel drug delivery system. The *in vitro* antitumor effects was studied in two kinds of gastric cancer cells and the *in vivo* antitumor effects was evaluated in orthotropic xenografts. Western blot and immunohistochemistry analyses were utilized in order to elucidate the possible mechanisms underlying the superior antitumor effect of the gelatin delivery system.

## Results

### Characterization of P/T-NPs-Gelatin

Gelatin has a phase-shift below and above its melting temperature of 35 °C. In the preparation process, the P/T-NPs solution was mixed with gelatin powders at 55 °C. The mixture of aqueous gelatin solution and P/T-NPs suspension was then stored at 4 °C, where it gradually transitioned into a hydrogel (P/T-NPs-Gelatin).

**Figure 1:**
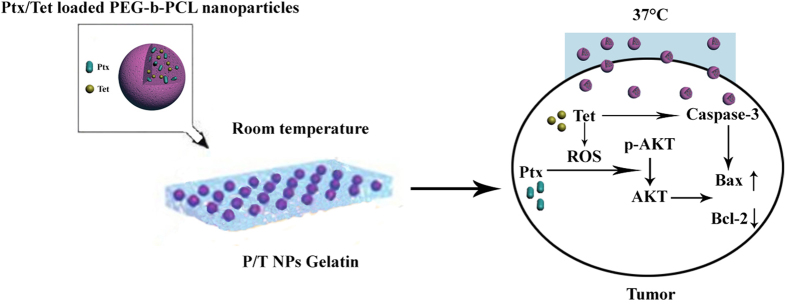
Schematic illustration of the possible mechanisms of P/T-NPs-Gelatin when implanted *in vivo*.

**Figure 2:**
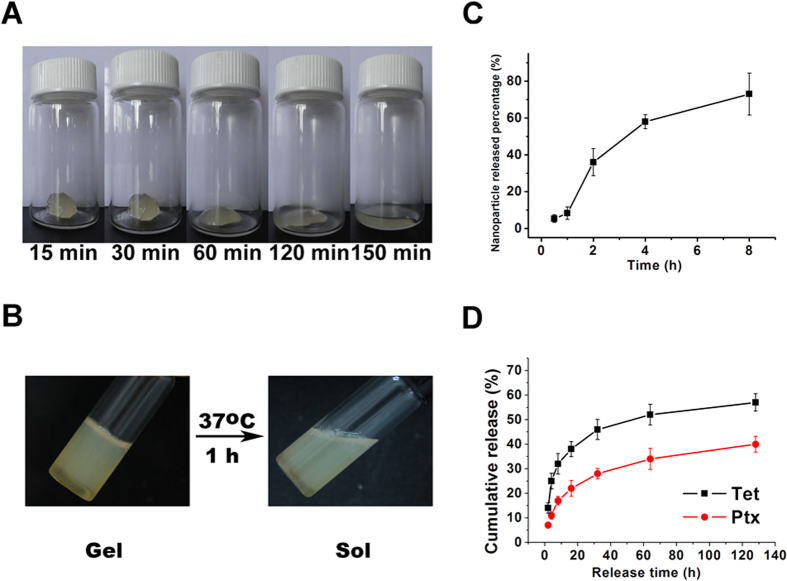
Characterization of P/T-NPs-Gelatin. (**A**) images of P/T-NPs-Gelatin when incubated at 37 °C for different time periods. (**B**) images of P/T-NPs-Gelatin in PBS solution that undergoes a gel sol transition at 37 °C. (**C**) *In vitro* release profile of P/T-NPs from gelatin in PBS at 37 °C. (**D**) *In vitro* release profiles of Ptx and Tet from P/T-NPs-Gelatin in PBS at 37 °C.

As shown in Fig. 2A,B, P/T-NPs-Gelatin melted slowly, within 150 min, with a high viscosity and poor liquidity at 37 °C, which indicated a phase change of P/T-NPs-Gelatin at body temperature (Fig. 2A,B). Meanwhile, a small amount of the melted gel was sampled and monitored by DLS. It was determined that the average size of loaded nanoparticles was approximately 70–90 nm, which was almost the same size as the nanoparticles before loaded into the gelatin hydrogel (Fig. 1S), implying that the encapsulated Ptx/Tet co-loaded PVP-b-PCL nanoparticles were released gradually without much damage.

The *in vitro* release curve of P/T-NPs from P/T-NPs-Gelatin at 37 °C is shown in Fig. 2C. It is shown that the release of P/T-NPs from the gelatin was accelerated with the gradual liquidity of the jelly. While less than 10% of the nanoparticles were released within the first hour, more than 70% of the nanoparticles were released by the time the gel-sol phase change was nearly complete. The drug release kinetics from the gelatin were studied by HPLC, as shown in Fig. 2D. It was observed that both Ptx and Tet were released from the gelatin in a sustained manner within 5 days. However, at the end of the 5-day period, nearly 50% of the two drugs still remained in the gelatin.

To further determine if there was any Tet or Ptx in gelatin instead of in the nanoparticles, ultra-high amount of gelatinase was prepared freshly and added into the gelatin gel containing drug loaded NPs. Fully degradation of the gel could be seen after only 15 mins. Then the drug loaded nanoparticles were harvested via centrifugation followed by the detection of the content of Ptx and Tet in the supernatant by HPLC. Since the treatment time is very short, drug release from the NPs was relatively limited. Drug in the supernatant could be safely ascribed to be loaded in the gel rather than in NPs in non-particular form. As shown in Fig. 2S, no observable characteristic peaks corresponding to PTX and Tet were detected, which demonstrated that the gel contained negligible amount of drug. On this basis, we assumed that all the drugs were loaded in NPs and the lower releasing rate of PTX and Tet from NPs might be responsible for the mismatched releasing profiles between drugs and NPs.

### *In vitro* antitumor effect of P/T-NPs-Gelatin against BGC-823 cells

The *in vitro* cytotoxicity of drug-loaded nanoparticles and gelatin was evaluated by MTT assay, with or without the presence of N-acetyl cysteine (NAC). NAC, an anti-oxidant and ROS scavenger, was employed to block the induction of ROS by drugs since ROS generation may be an important factor in the synergistic antitumor effect of the drugs. Less than 10% of cells died when exposed to a high concentration of empty nanoparticles (500 μg/ml) and gelatin. In addition, we tested a series of NAC concentrations and determined 500 μM to be the highest without causing toxicity to the BGC-823 cells (data not shown).

**Figure 3:**
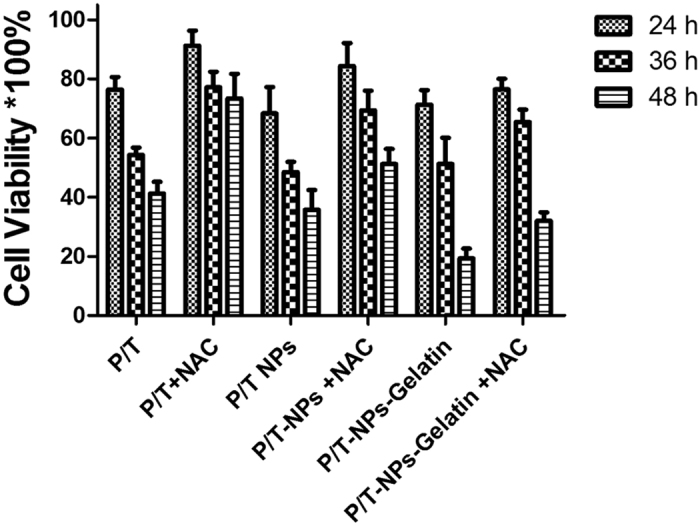
*In vitro* cytotoxicity of Ptx and Tet in different formulations against BGC-823 cells with or without the presence of N-acetyl cysteine (NAC).

It is shown in Fig. 3 that the free-drug combination (Ptx/Tet), P/T-NPs, and P/T-NPs-Gelatin all inhibited the proliferation of BGC-823 cells in a time-dependent manner. The values of IC50s for Ptx/Tet combination were listed in Table 1. However, the *in vitro* cytotoxicity of both P/T-NPs and P/T-NPs-Gelatin was much stronger than that of the free-drug combination. The cytotoxicity of P/T-NPs-Gelatin increased significantly faster than P/T-NPs. For example, cell viability decreased from approximately 70% to 35% after an additional 24 h of incubation time (from 24 h to 48 h). The gelatin led to a much greater reduction in cell viability, from more than 70% to less than 20%, which indicates the sustained inhibition of cell proliferation through its controlled release. Most importantly, NAC greatly protected the cell from the cytotoxicity of the free-drug combination (P/T). In contrast, it moderately reversed the cytotoxicity of P/T-NPs, while there was less protection by NAC in the cytotoxicity of P/T-NPs-Gelatin. To better show the reliability of the results, we repeated the cytotoxicity test on another gastric cancer cell line SGC-7901, which showed the same trend as on BGC-823 cells (Fig. 3S and Table 2 in supplementary information file).

**Figure 4:**
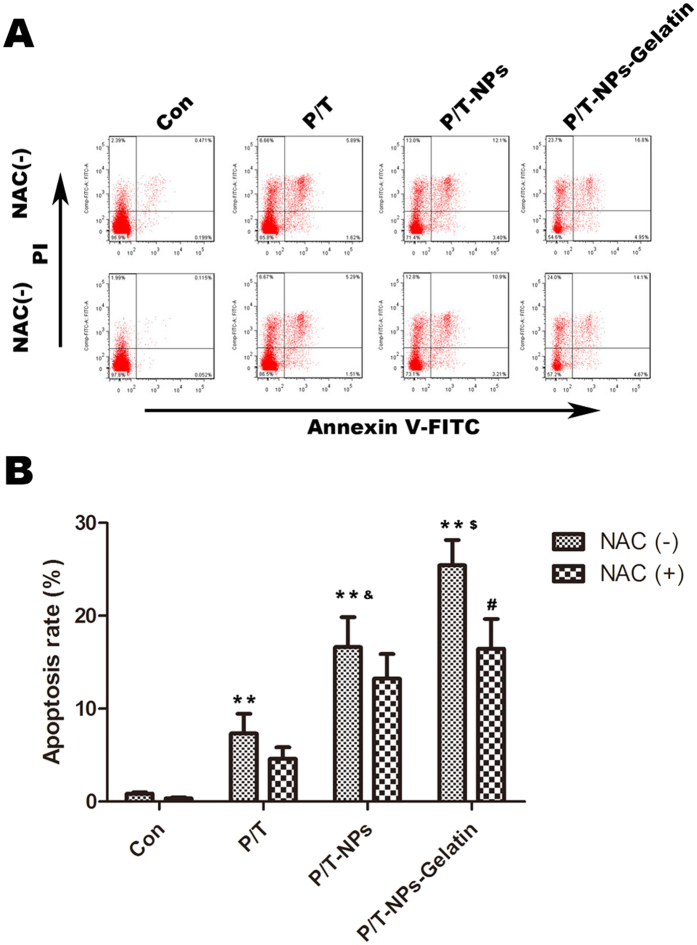
Induction of apoptosis by Ptx and Tet in different formulations with or without the presence of N-acetyl cysteine (NAC) detected by FACS. (**A**) Apoptosis of cells detected by FACS when exposed to different agents for 48 h. (**B**) Quantification of apoptosis rate based on FACS results. ^**^represents p < 0.01 vs control group. ^$^represents p < 0.01 vs the corresponding group treated with P/T-NPs. & represents p < 0.01 vs the corresponding group treated with P/T. ^#^represents p < 0.05 vs the corresponding group of P/T-NPs-Gelatin without the co-treatment of NAC.

The apoptosis of BGC-823 cells was quantified by fluorescent Annexin V-FITC/PI double staining with Flow cytometry. Figure 4 indicates that P/T-NPs-Gelatin induced significantly more apoptosis of BGC-823 cells than free drug combination (P/T) or delivered by nanoparticles (P/T-NPs) (p < 0.01). In addition, NAC co-treatment significantly protected cells from apoptosis induced by P/T-NPs-Gelatin (p < 0.05). The same trend could be found in the results of SGC-7901 cells (Fig. 4S in supplementary information file).

**Figure 5:**
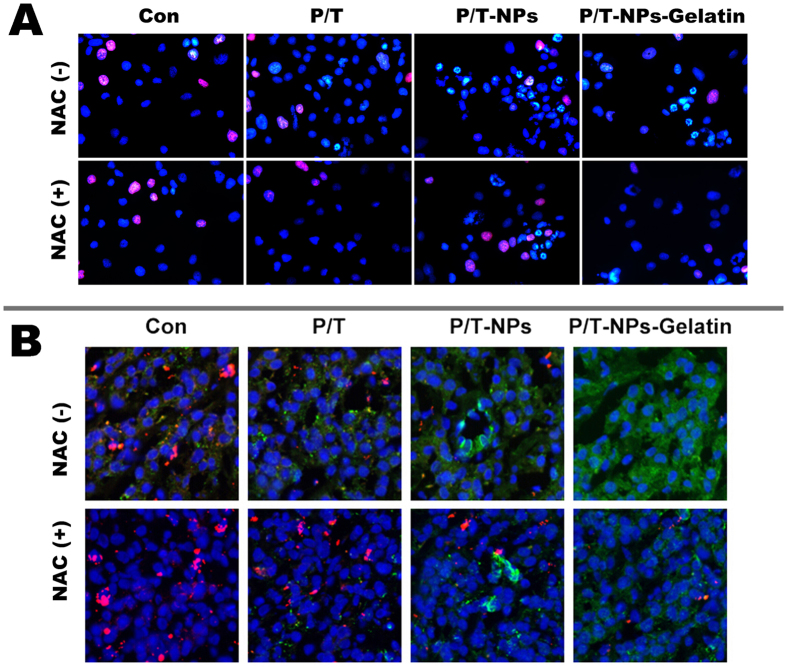
Measurement of cell proliferation and ROS induction in BGC-823 cells by Ptx and Tet in different formulations with or without the presence of N-acetyl cysteine (NAC). (**A**) Edu staining of BGC-823 cells exposed to equivalent doses of Ptx/Tet, P/T-NPs or P/T-NPs-Gelatin for 48 h. (**B**) Ki-67 and cleaved caspase-3 immunofluorescence of BGC-823 cells exposed to equivalent doses of Ptx/Tet, P/T-NPs or P/T-NPs-Gelatin for 48 h.

In addition, cell proliferation was evaluated by Edu staining (Fig. 5A). It showed the same trend as in cytotoxicity test. Among the four groups without the presence of NAC, the least Edu staining was observed in the group of P/T-NPs-Gelatin treatment, which demonstrated that P/T-NPs-Gelatin induced the strongest inhibition of the proliferation in BGC-823 cells. In addition, NAC partially protected the cells to the anti-proliferative effect of P/T-NPs-Gelatin.

To further evaluate the cell proliferation and apoptosis, we performed dual fluorescent staining of ki-67 and cleaved caspase-3. As shown in Fig. 5B, the least expression of ki-67 (red fluorescence) and the most expression of cleaved caspase-3 (green fluorescence) were observed in cell treated with P/T-NPs-Gelatin, which clearly proved that P/T-NPs-Gelatin was the most effective agent to inhibit cell proliferation and induce the apoptosis.

**Figure 6:**
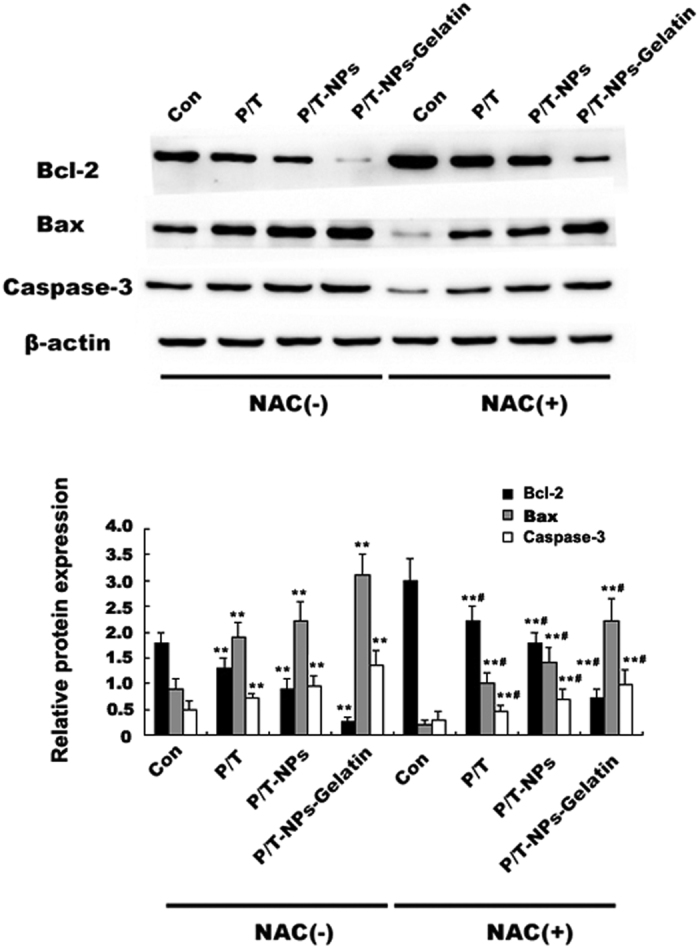
The expression of Bcl-2, Bax and Caspase-3 in cells treated with equivalent doses of Ptx and Tet in different formulations with or without the presence of N-acetyl cysteine (NAC).

The expression of apoptotic proteins (Bcl-2, Bax, and Caspase-3) were evaluated by western blot (Fig. 6). P/T-NPs-Gelatin was more effective in inhibiting anti-apoptotic Bcl-2 and activating pro-apoptotic Bax than the other two formulations of Ptx and Tet, which led to an increase of apoptosis. In addition, NAC partially reversed this kind of effect.

### Superior inhibitory effect of P/T-NPs-Gelatin on the migration and invasion of BGC-823 cells

**Figure 7:**
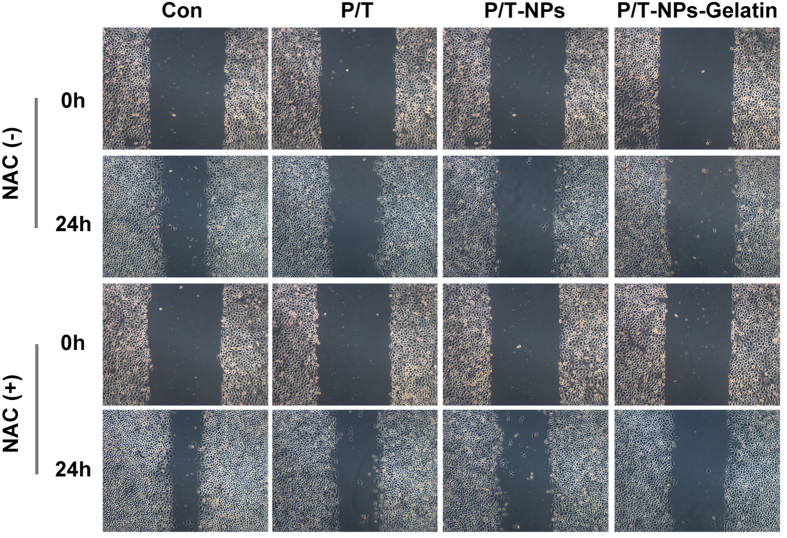
Migration ability of BGC-823 cells exposed to equivalent doses of Ptx and Tet in different formulations with or without the presence of N-acetyl cysteine (NAC). Phase micrograph of BGC-823 cells at 0 h or 24 h after monolayer wounding.

The wound scratch assay was utilized to evaluate the influence of different agents on the ability of cells to migrate. It is shown in Fig. 7 that delivery of Ptx and Tet, freely or in both nanoparticles and gelatin, inhibited the migration of BGC-823 cells. Moreover, drugs delivered by gelatin significantly impaired the ability of cells to migrate compared with the corresponding concentration of free drugs or nanoparticles. Most importantly, the presence of NAC partially counteracted the inhibitory effect on migration when cells were exposed to different preparations of Ptx and Tet.

**Figure 8:**
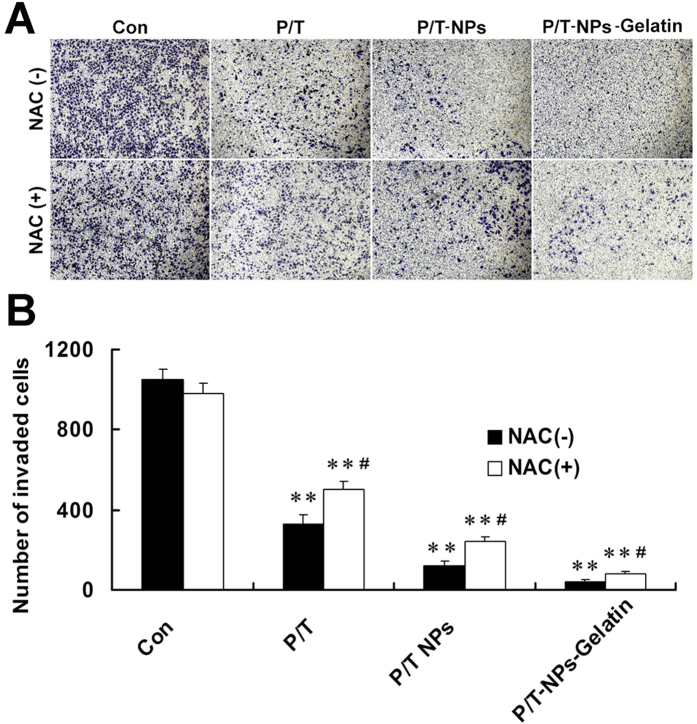
Invasive ability of BGC823 cells treated by P/T-NPs-Gelatin with or without the presence of N-acetyl cysteine (NAC). (**A**) Cell invasive ability of BGC823 cells exposed to equivalent doses of Ptx/Tet, P/T-NPs or P/T-NPs-Gelatin for 24 h. (**B**) Quantification of cell invasion. Each data point represents the mean ± SD from three independent experiments; ^**^represents p < 0.01 versus control. ^#^represents p < 0.05 versus the corresponding group without the presence of NAC.

The invasive ability of BGC-823 cells was evaluated by transwell assay. As indicated in Fig. 8, cells stained by crystal-violet represented successfully invading cells through Matrigel-coated filters. It was demonstrated that cells treated with P/T-NPs-Gelatin showed the weakest ability to infiltrate as supported by the least amount of stained cells, which could be partially reversed by the antioxidant NAC. Quantitative analysis showed that the number of successfully invading cells in the P/T-NPs-Gelatin group was the least among the four groups and that NAC could partially attenuate this effect.

### Superior *in vivo* antitumor effect of P/T-NPs-Gelatin in orthotropic model

**Figure 9:**
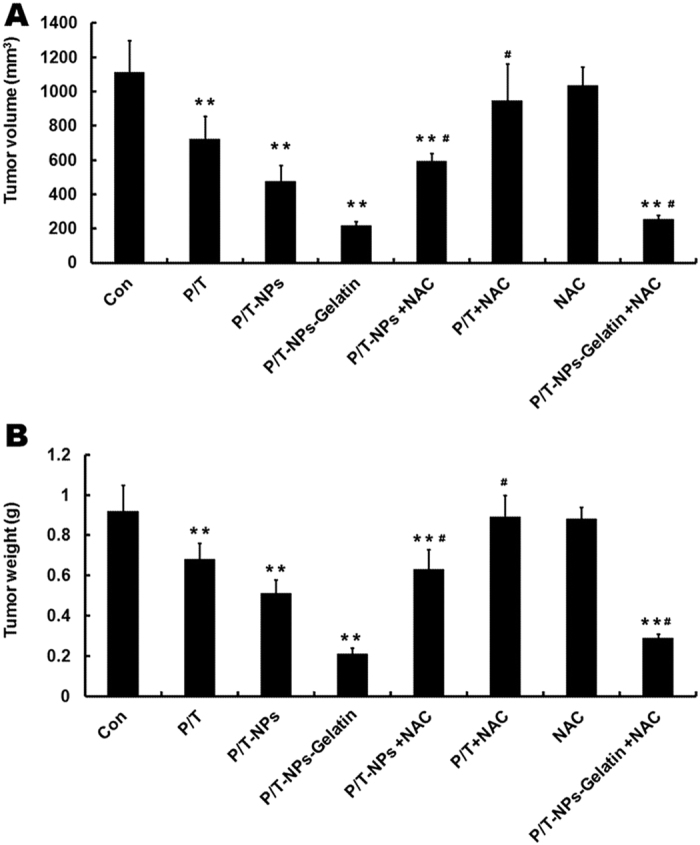
Tumor volume (**A**) and weight (**B**) of mice receiving different formulations of Ptx and Tet at the end of experiment. Each data point represents the mean ± SD; ^**^represents p < 0.01 versus control. ^#^represents p < 0.05 versus the corresponding group without the presence of N-acetyl cysteine (NAC).

In the current study, mice were divided into eight groups and then treated with different regimens. Tumor nodules were extracted and measured at the end of the experiment. We evaluated the antitumor effect of the combination of Ptx and Tet in different formulations, with or without the co-treatment of NAC, in this orthotropic model. As shown in Fig. 9A, the combination of Ptx and Tet in all formulations without the co-delivery of NAC, exhibited significant antitumor effects as compared to the control group (p < 0.01). Moreover, P/T-NPs-Gelatin retarded the orthotropic tumor growth most significantly among the treated groups. The volume of the tumor nodule from the mice that received a combination of Ptx and Tet freely, in nanoparticles, or in gelatin, without NAC administration, was 723 ± 132 mm^3^, 495 ± 95 mm^3^, and 217 ± 26 mm^3^, respectively. Similarly, the tumor weights of the three formulations of Ptx and Tet were 0.68 ± 0.08 g, 0.51 ± 0.07 g, and 0.21 ± 0.03 g, respectively (Fig. 9B). Both the volume and the weight of the orthotropic tumor in mice that received P/T-NPs-Gelatin was the smallest and lightest among the treated groups.

In addition, we also evaluated whether NAC could influence the antitumor effects of the three Ptx and Tet formulations. NAC alone had nearly no proliferative or inhibitory effect on tumor growth. However, NAC co-treatment with the different drug formulations partially counteracted their antitumor effects. NAC reversed the growth inhibitory effect of the free Ptx and Tet combination greatly, as shown by the mean tumor volume increasing from 723 mm^3^ to 947 mm^3^. In contrast, NAC more moderately counteracted the effect of Ptx and Tet delivered in nanoparticles, with the mean volume increasing from 475 mm^3^ to 592 mm^3^ (p < 0.05 vs free Ptx and Tet). Most importantly, P/T-NPs-Gelatin showed significant resistance to the reversal effect of NAC, with the mean volume slightly increasing from 217 mm^3^ to 254 mm^3^ (p < 0.05 vs Ptx and Tet in nanoparticles). The trend of tumor weight also led to the same observations.

### The expression of relative proteins in orthotropic xenografts treated with different formulations of Ptx and Tet

To further elucidate the possible mechanisms underlying the superior antitumor effect of P/T-NPs-Gelatin, we measured the expression of relative proteins in xenograft tumors by western blot and immunohistochemistry analysis.

**Figure 10:**
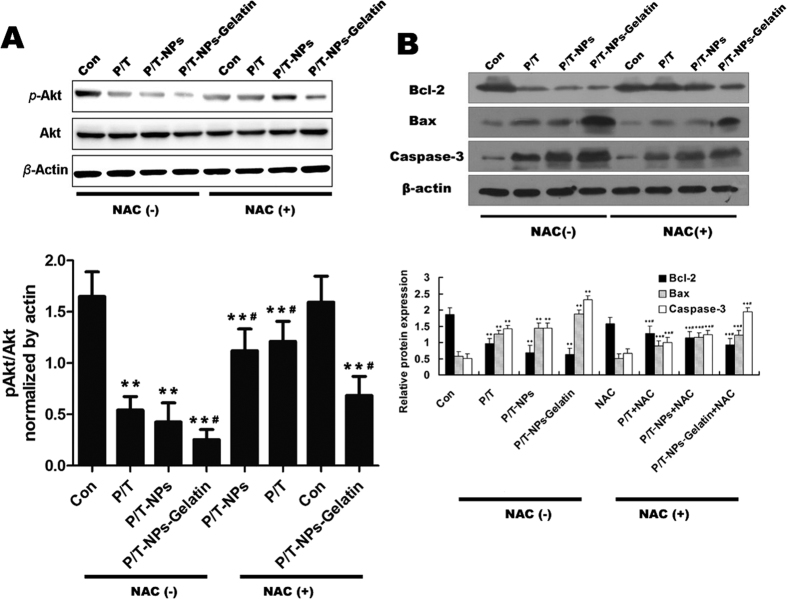
(**A**) the expression of p-Akt and Akt in tumors extracted from different groups of mice. Semi-quantification of the gel image, normalized to β-actin control. Each data point represents the mean ± SD; ^**^represents p < 0.01 versus control. ^#^represents p < 0.05 versus the corresponding group without the presence of N-acetyl cysteine (NAC). (**B**) The expression of Bcl-2, Bax and Caspase-3 in tumors extracted from different groups of mice. Semi-quantification of the gel image, normalized to β-actin control. Each data point represents the mean ± SD from three independent experiments; ^**^represents p < 0.01 versus control. ^#^represents p < 0.05 versus the corresponding group without the presence of NAC.

As indicated in Fig. 10A, different formulations of Ptx and Tet showed varied influences on the expression of phospho-Akt (p-Akt). It is noted that without the presence of NAC, Ptx and Tet, whether delivered freely, in nanoparticles, or in gelatin, weakened the phosphorylation of Akt. The expression of p-Akt in BGC-823 cells treated with both P/T-NPs and P/T-NPs-Gelatin was significantly less than in the control group. Moreover, delivery of the drugs by gelatin produced the greatest inhibition of Akt phosphorylation among the three drug formulations. We then examined the effect of NAC on the expression of p-Akt and found that Akt repression by P/T, P/T-NPs, and P/T-NPs-Gelatin was partially reversed by co-treatment with NAC. This finding indicated that the antioxidant NAC could protect the cells from apoptosis by scavenging drug-induced intracellular ROS.

The expression of apoptotic proteins was then measured by western blot analysis (Fig. 10B). The P/T-NPs-Gelatin induced the expression of Bax and reduced the expression of Bcl-2 more efficiently than the equivalent dose of P/T or P/T-NPs, thereby leading to a significant increase in the expression of activated Caspase-3. NAC co-treatment partially reversed the apoptotic induction of the three types of drug formulations, indicating that the drug-induced apoptosis was activated by the increased intracellular ROS level.

**Figure 11:**
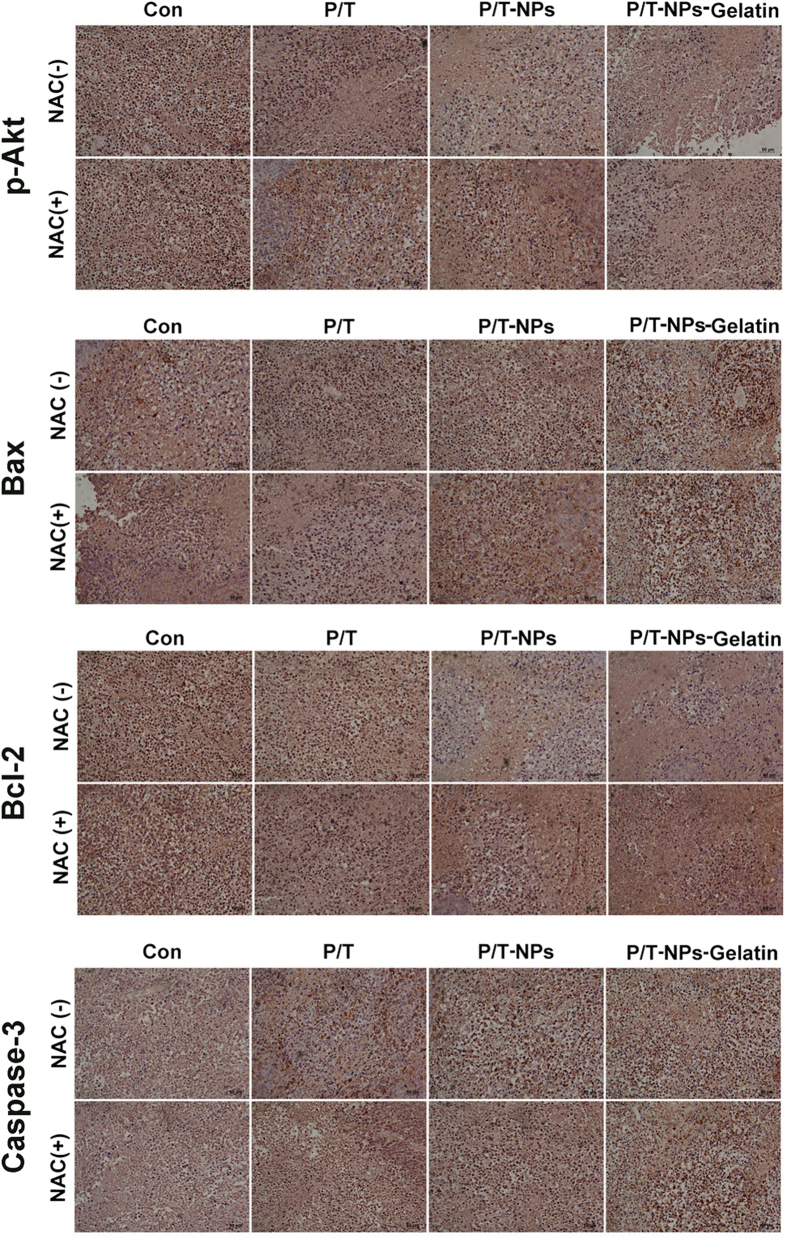
Immunohistochemistry of p-Akt, Bax, Bcl-2, and Caspase-3 in tumors extracted from different groups of mice.

IHC staining revealed the same trend as the western blot analysis. As shown in Fig. 11, positive staining of p-Akt, Bax, Bcl-2, and Caspase-3 was mainly located in cytoplasm and cell membrane. P/T-NPs-Gelatin caused a greater attenuation in the expression of p-Akt than the P/T or P/T-NPs groups. There was significantly less positive staining for p-Akt in xenograft tumors treated with P/T-NPs-Gelatin rather than P/T or P/T-NPs. The expression of apoptosis-related proteins, analyzed by IHC analysis, also demonstrated the superior apoptosis-induced effect of P/T-NPs-Gelatin. A more prominent increase in positive cell staining of Bax was accompanied by a much greater decrease in Bcl-2 in P/T-NPs-Gelatin group. Cells that stained positive for activated Caspase-3, the marker of apoptosis, underwent a noticeable increase in expression in the P/T-NPs-Gelatin group, thus supporting the finding that P/T-NPs-Gelatin could efficiently delay tumor growth through the induction of apoptosis.

## Discussion and Conclusion

Ptx has been used for decades as one of the main components of first-line chemotherapy treatment for gastric cancer, but its application has been substantially counteracted by severe side effects and emerging chemoresistance in the clinic^20^. Therefore, the development of promising chemo-sensitizing strategies utilizing a combination of several anticancer drugs is urgently needed, as this is a proven strategy for effective therapy.

In our previous study, we demonstrated that co-delivery of Tet and Ptx in mPEG-PCL nanoparticles not only is more stable than Ptx-loaded nanoparticles but also capitalizes on the synergistic anticancer efficiency of Tet and Ptx^10^,^16^. In the current study, the antitumor effects of Ptx and Tet were evaluated by delivering nanoparticles and gelatin with free drugs as a control. The *in vitro* cytotoxicity results measured by both the MTT assay and FACS demonstrated that there was a significant difference between the apoptosis rates induced by P/T-NPs-Gelatin and P/T-NPs. Moreover, P/T-NPs-Gelatin was more resistant to the reversal effect of the antioxidant agent, NAC, than P/T-NPs or P/T. Similarly, P/T-NPs-Gelatin more strongly inhibited cell migration and invasion than P/T-NPs or P/T. The superior results of P/T-NPs-Gelatin may be due to the controlled release of the drugs. During the preparation process, the Ptx and Tet co-loaded mPEG-PCL nanoparticles were further encapsulated within gelatin, which generated a solid jelly that could gradually melt to sol liquid at 37 °C. As shown in Fig. 2C,D, P/T-NPs were released in a sustained manner based on the degradation of gelatin. Therefore, controlled release of drug-loaded nanoparticles from the gelatin during the melting process contributed most to the sustained release of the loaded drug and enabled the continuous exposure to the encapsulated drugs.

In the *in vitro* cytotoxicity test and apoptosis detection by Flow cytometry, there was a discrepancy between cell viability and apoptosis for P/T and P/T-NPs groups for NAC (-). To our understanding, change of cell viability can be influenced by multiple factors including the proliferation and apoptosis of cells. Therefore, the different anti-proliferative and apoptosis induction effects of P/T or P/T-NPs may lead to the discrepancy between the viability and apoptosis. In addition, apoptosis detected by FACS included early apoptosis and late apoptosis. Cells in the stage of early apoptosis are still alive and can react with MTT while late apoptotic cells are necrotic cells which will not react with MTT. In this case, the different proportion of early and late apoptotic cells may result in the above-mentioned discrepancy.

Malignant tumors are characteristically invasive and metastasizing, which often leads to the failure of chemotherapy^21^. Therefore, it is important to inhibit the invasion and metastasis of the tumor through each step of the process, including degradation of the extracellular matrix, penetration of the vascular endothelial cells, intravascular circulation, and adhesion elsewhere in the body^22^. The ability of cancer cells to migrate and invade is essential to the entire progression process^23^. Cells from BGC-823, a poorly differentiated adenocarcinoma cell line, possess high invasive and penetrating potential^24^. As a result, the therapeutic efficacy of drugs is closely related to the restriction of cell migration and invasion.

During the *in vivo* evaluation, we established an orthotropic gastric cancer model by reproducing the organ environment where human tumors grow, in order to mimic the human tumor microenvironment more completely^25^,^26^. P/T-NPs-Gelatin were implanted directly on the surface of orthotropic tumor tissues via laparotomy. As indicated in Fig. 1, antitumor evaluation *in vivo* showed that the implanted P/T-NPs-Gelatin delayed orthotropic tumor growth more significantly than intraperitoneal administration of P/T-NPs. Intraperitoneal delivery of P/T-NPs was originally chosen as a therapeutic regimen because it was believed that intraperitoneal delivery could reach a relatively high drug concentration for treating peritoneal surface malignancies and regional recurrence of gastrointestinal cancer. Figure 9 shows that although P/T-NPs showed stronger *in vivo* efficacy than the free combination of Ptx and Tet, the inhibitory growth effect of P/T-NPs was still significantly weaker than that of P/T-NPs-Gelatin. Most importantly, P/T-NPs-Gelatin showed a much greater resistance to the reversal effect of the antioxidant NAC.

This resistance may result from the characteristic pharmacokinetics of the implanted P/T-NPs-Gelatin. Implanting P/T-NPs-Gelatin on the surface of the tumor led to it gradually melting into a viscous sol at body temperature, as shown in Fig. 2. After transforming into a sol *in vivo*, P/T-NPs-Gelatin not only released the encapsulated P/T-NPs but also formed a nanoparticle depot in the peritumoral region. The nanoparticles allowed for continuous diffusion of the drug into the tumor and led to a steadily increasing local drug concentration.

The imbalance of intracellular redox levels inside the cancer cells was a key factor influencing the cytotoxicity of Ptx^27^. Tumor cells can survive and proliferate by developing an enhanced anti-oxidative system as a result of accelerated cell metabolism^5^. It has been previously reported that the cytotoxicity of Ptx could be reversed by treatment with antioxidant agents^6^. Data from the current study also demonstrated that NAC supplementation could attenuate the efficacy of the combined delivery of Ptx and Tet to some extent. In addition, it was also shown that the nano-formulation of Ptx and Tet, especially P/T-NPs-Gelatin, was highly resistant to the reversal effect of NAC, thereby indicating that the considerably prolonged release of the loaded drugs counteracted the protective effect of NAC against apoptosis.

The other factor related to Ptx cytotoxicity was the enhanced expression of the survival pathway, PI3K/Akt^28^,^29^. Previous studies have reported that the suppression of Akt pathway activation is of great significance to the cytotoxicity of Ptx^30^,^31^. There was evidence that the ROS scavenger could counteract the cytotoxicity of Ptx and that the underlying mechanism was associated with the alteration of cellular total anti-oxidative capacity in numerous cancer cells^6^,^32^,^33^. In contrast, inducing over-production of intracellular ROS by Tet could greatly enhance the apoptosis of cancer cells by Ptx, which has been demonstrated in our previous research^10^. As reported in previous studies, intracellular ROS accumulation, sequential Akt pathway inactivation, and the subsequent adjustment of apoptotic-regulated proteins can trigger the apoptotic cascade^10^,^34^. *In vitro* and *in vivo* data (Figs 6 and 10) shows that P/T-NPs-Gelatin significantly repressed the activation of p-Akt, causing inhibition of the downstream anti-apoptotic Bcl-2 proteins and induction of the pro-apoptotic Bax proteins. This result was in accordance with the results from IHC analysis (Fig. 11). Moreover, the molecular modulating effect of P/T-NPs-Gelatin on related proteins varied slightly under the influence of NAC, which coincided with what was observed in the tumor volumes and weights.

In the current study, we report an efficient, localized therapeutic strategy employing delivery of co-loaded Tet and Ptx by a nanoparticle-loaded gelatin system (P/T-NPs-Gelatin). Tet and Ptx loaded mPEG-PCL nanoparticles (P/T-NPs) were encapsulated into the physically cross-linked gelatin hydrogel and then implanted on the tumor site for continuous drug release. The drug-loaded gelatin hydrogel underwent a phase change when temperatures increased, during which encapsulated nanoparticles and drugs were released in a sustained manner. P/T-NPs-Gelatin inhibited the growth, migration and invasion of BGC-823 cancer cells more effectively than the other groups, which partially counteracted the reversal effect of NAC. P/T-NPs-Gelatin was shown to be the most useful therapeutic regimen with the lowest tumor weight and volume. In addition, P/T-NPs-Gelatin significantly repressed the activation of p-Akt, thereby regulating apoptotic proteins. Therefore, local implantation of P/T-NPs-Gelatin may be a promising therapeutic strategy for the treatment of gastric cancer.

## Materials and Methods

### Materials

Ptx and Tet were purchased from Sigma-Aldrich (St Louis, MO). Type-B gelatin (225 bloom strength), with 100–115 mmol of carboxylic acid per 100 g of protein, an isoelectric point of 4.7–5.2, and an average molecular weight of 40–50 kDa, was purchased from Sigma-Aldrich (St Louis, MO). Polyethylene glycol (PEG) at a molecular weight of 4 kd was purchased from Sigma-Aldrich (St Louis, MO). ε-Caprolactone (ε-CL, Sigma) was dehydrated by CaH_2_ at room temperature and distilled under reduced pressure. 1-(4,5-Dimethylthiazol-2-yl)-3,5-diphenylformazan (MTT) was purchased from Sigma-Aldrich. All remaining reagents were of analytical grade and used without further purification.

The human low-differentiated gastric adenocarcinoma cell line, BGC-823 and SGC-7901, was purchased from the Shanghai Institute of Cell Biology (Shanghai, China) and cultured in RPMI 1640 medium with 10% fetal bovine serum and 100 U/ml penicillin-streptomycin at 37 °C in a humidified atmosphere with 5% CO_2_.

Six to eight-week-old BALB/c athymic nude mice were purchased from the Shanghai SLAC Laboratory Animal Co., Ltd. (Shanghai, China). The mice were housed and maintained in the animal facility of the Animal Center of Nanjing Medical University. The animal protocol was reviewed and approved by the Animal Care and Use Committee of Nanjing Medical University.

### Preparation of P/T-NPs-Gelatin

Ptx/Tet co-loaded PEG-b-PCL gelatin hydrogel (P/T-NPs-Gelatin) was prepared through a two-step process. mPEG-b-PCL block copolymers were synthesized by a ring opening copolymerization as previously described^10^. Ptx/Tet co-loaded nanoparticles (P/T-NPs) were then prepared by a nano-precipitation method as previously described with some minor modifications^10^. Briefly, 20 mg PEG-b-PCL block copolymers and a predetermined amount of Tet and Ptx were first dissolved together in an aliquot of acetone, after which this solution was added dropwise into 10 volumes of double distilled water with mild agitation at room temperature. The solution was dialyzed in a dialysis bag (MWCO 12 kDa) to remove acetone thoroughly. The resulting solution was filtered through a 0.22 μm filter to remove non-entrapped drugs and adjusted to 10 mL as stock solution for hydrogel preparation. Then, 500 mg of gelatin powder was added to 3 mL of nanoparticle stock solution and then quickly dissolved at 55 °C for 10 min to form a viscous mixture, resulting in Ptx and Tet concentrations of 0.5 mg/mL. The desired amount of the resulting solution was cast into a plastic 24-well cell culture plate, after which the hydrogel was fabricated and stored at 4 °C until use. The final form of the hydrogel was carefully cut to make the appropriate dimension according to the drug loading (eg. 0.5 cm long, 0.5 cm wide and 0.2 cm high) for future use.

### Phase change and *in vitro* nanoparticle release from the P/T-NPs-Gelatin

To examine the *in vitro* physical state, stability, and aqueous solubility of the P/T-NPs-Gelatin at 37 °C, the hydrogel was placed into a 1 mL PBS solution, stored at 37 °C for up to 2 h, and then imaged at different time points. The prepared P/T-NPs-Gelatin (made of 1 mL mixture) was placed into 1 mL of PBS in an eppendorf tube and maintained at 37 °C. The released nanoparticles in the supernatant was collected by centrifuging (3500 rpm, 15 min) and then weighted at each time point. The percentage of nanoparticles released was determined by the following equation: release percentage = weight of released nanoparticles/weight of initial nanoparticles in gelatin ×100%.

### *In vitro* Tet and Ptx release from the P/T-NPs-Gelatin

The drug release profiles of Tet and Ptx from the hydrogels was examined. Briefly, P/T-NPs-Gelatin, made of a 1 mL mixture, was inserted into a dialysis bag (MWCO 12 kD). The dialysis bag was then immersed in 20 mL of PBS at 37 °C. At predetermined time points, a certain amount of PBS was collected for HPLC analysis, and an equal amount of liquid was added in. The concentration of Tet and Ptx was analyzed on a Shimadzu HPLC system, as reported in our previous study.

### *In vitro* cytotoxicity studies of P/T-NPs-Gelatin

The combination of free Ptx and Tet were dissolved in DMSO and then diluted in Medium with the concentration of DMSO being 0.05%. Lyophilized powder of P/T-NPs were dissolved in Medium to achieve the desired concentration. P/T-NPs-Gelatin was maintained at 37 for 1 h to change into a viscous sol before incubated with the cells and then diluted in Medium. BGC-823 cells were incubated with P/T-NPs-Gelatin, P/T-NPs, and a combination of Ptx and Tet, with or without the presence of the antioxidant NAC for different intervals. DMSO treatment (concentration = 0.05%) was performed simultaneously as solvent control. The concentration of Ptx and Tet were 100 nM and 10 μM, respectively, while the concentration of NAC was 400 μM. For each treatment, 5 replicates were measured to provide the most reliable results. Cell viability was measured using the MTT assay, as reported in our previous study^10^. The same cytotoxicity test was performed in SGC-7901 as well.

### Flow cytometry analysis

Cells were treated with different agents at the same concentration in cytotoxicity test for 48 h and then stained with double staining of Annexin V-FITC/PI (Molecular probes, Eugene, OR, USA). 1X Annexin-binding buffer was made by adding 1 mL 5X Annexin-binding buffer (Component C) to 4 mL deionized water. A 100 μg/mL working solution of PI was made by diluting 5 μL of the 1 mg/mL PI stock solution (Component B) in 45 μL 1X Annexin-binding buffer. The stained cells were analyzed by FACScan flow cytometer (Becton Dickinson, CA, USA).

### Edu Staining for cell proliferation

BGC-823 cells were treated with different agents at the same concentration in cytotoxicity test. After 48 h, cells were washed by PBS and stained with Edu (50 μM) solution for 2 h according to the protocol. After that, cells were stained with 300 nM DAPI for 5 mins in the dark. Then cells were washed and imaged under a fluorescent microscopy with an excitation/emission wavelength of 350/550 nm (Olympus, Japan). The proliferation of cells were measured by counting the Edu stained cells (red) in ten different fields.

### Dual immunofluorescent staining for proliferation and apoptosis

Briefly, BGC-823 cells were seeded in 6 wells plate at a density of 1 × 10^6^ and incubated for a certain time period in order to form a confluent cell monolayer. After 24 h, cells were treated with different agents at the same concentration in cytotoxicity test for 48 h. The cells were fixed with 4% formaldehyde for 10 minutes at room temperature and blocked with PBS containing 10% goat serum, 0.3 M glycine, 1% BSA and 0.1% tween for 2h at room temperature. Staining of the treated cells with anti-ki-67 and anti-cleaved caspase-3 (Abcam, Cambridge, MA) was performed overnight at 4 °C in PBS containing 1% BSA and 0.1% tween. Cells were then incubated with the mixture of two secondary antibodies in 1% BSA for 1 hr at room temperature in dark. Nuclei were counterstained with DAPI and are shown in blue. Cells were then washed three times with 1× PBS and samples were sealed under coverslips. Cells were examined using a confocal microscope (Carl Zeiss Jena, Oberkochen, Germany).

### Wound scratching assay

An *in vitro* wound scratching assay was applied to measure the cell migration of BGC-823 cells under the treatment of different agents. BGC-823 cells were seeded in a 6-well plate and incubated for a certain time period in order to form a confluent cell monolayer. After 12 h in serum-free culture, a wound was inflicted on the cells by using a sterile pipette to scratch the monolayer, and pictures were taken using a microscope. At this point, cells were exposed to different types of agents at the same concentration as in the cytotoxicity test. After 24 h, cells were photographed again under the same conditions.

### Transwell invasion assays

The ability for drug-loaded nanoparticles and hydrogels to infiltrate cells was evaluated using a transwell invasion assay. Briefly, BGC-823 cells were seeded in 24-well transwell chambers (Corning, Acton, MA, USA) and cultured without serum for 24 h. After digesting with trypsin, the cells were washed and added into the transwell chamber with serum-free RPMI 1640, while complete medium was used in the lower chamber as a chemo-attractant. Cells were then exposed for 48 h to the same concentrations of agents as in the cytotoxicity test. Pictures were taken of cells successfully invading the lower chamber of the transwell, from which the number of invading cells was then calculated.

### Animal Studies

The study protocol was approved by the Animal Use Committee of Nanjing Medical University and was carried out in accordance to with the guideline of experimental animals of Nanjing Medical University. Nude mice were purchased and then caged in groups of six. The mice were anesthetized using chloral hydrate, and 1 × 10^6^ BGC-823 cells were injected into the outside wall of the stomach. When the tumors had grown to approximately 7–10 mm^3^ after 7 to 10 days, the mice were divided into eight groups as follows: (1) control; (2) intraperitoneal administration of NAC at a dose of 100 mg/kg; (3) intraperitoneal administration of Ptx and Tet at a dose of 7.5 mg/kg and 15 mg/kg, respectively; (4) intraperitoneal administration of P/T-NPs at the equivalent dose; (5) intraperitoneal administration of P/T together with NAC; (6) intraperitoneal administration of P/T-NPs together with NAC; (7) implantation of P/T-NPs-Gelatin in the tumor site at the equivalent dose; and (8) implantation of P/T-NPs-Gelatin in the tumor site at the equivalent dose together with the intraperitoneal administration of NAC. For the implantation of P/T-NPs-Gelatin, the required amount of dosing was calculated according to the weight of each mouse. Then the predetermined amount of Ptx and Tet was encapsulated into the gels and the desired size of P/T-NPs-Gelatin was made by casting the gels into required dimension. At the end of the 35-day experiment, the mice were sacrificed and the tumors were excised, weighed, and then either preserved in 4% buffered formalin for paraffin fixation or stored in liquid nitrogen for protein examination.

### Western blot analysis and Immunohistochemistry staining

The expression of related proteins extracted from tumor cells and tissues were measured using western blot analysis. Tumor tissues from the mice in various groups were extracted and prepared for western blot analysis or immunohistochemistry staining, as reported in our previous study^8^,^10^. The primary antibodies used were anti-p-Akt (SER 473), anti-Akt, anti-Bcl-2, anti-Caspase-3, and anti-β-actin (Cell Signaling Technology, Inc., Beverly, MA, USA).

### Statistical analysis

All data are presented as the means ± SD and analyzed for significant differences using a Student’s t-test or one-way ANOVA with SPSS 11.5 software. Significance was accepted at the 0.05 level of probability.

## Additional Information

**How to cite this article**: Zhang, H. *et al.* Efficient antitumor effect of co-drug-loaded nanoparticles with gelatin hydrogel by local implantation. *Sci. Rep.* **6**, 26546; doi: 10.1038/srep26546 (2016).

## Supplementary Material

Supplementary Information

## References

[b1] Siegel R., Ma J., Zou Z. & Jemal A. Cancer statistics, 2014. CA: a cancer journal for clinicians 64, 9–29, 10.3322/caac.21208 (2014). 24399786

[b2] Yuan M., Yang Y., Lv W., Song Z. & Zhong H. Paclitaxel combined with capecitabine as first-line chemotherapy for advanced or recurrent gastric cancer. Oncology letters 8, 351–354, 10.3892/ol.2014.2131 (2014). 24959275 PMC4063573

[b3] Landry W. D. & Cotter T. G. ROS signalling, NADPH oxidases and cancer. Biochemical Society transactions 42, 934–938, 10.1042/BST20140060 (2014). 25109982

[b4] Paul S., Sengupta S., Bandyopadhyay T. K. & Bhattacharyya A. Stevioside induced ROS-mediated apoptosis through mitochondrial pathway in human breast cancer cell line MCF-7. Nutrition and cancer 64, 1087–1094, 10.1080/01635581.2012.712735 (2012). 23061910

[b5] Laurent A. *et al.* Controlling tumor growth by modulating endogenous production of reactive oxygen species. Cancer research 65, 948–956 (2005). 15705895

[b6] Ramanathan B. *et al.* Resistance to paclitaxel is proportional to cellular total antioxidant capacity. Cancer research 65, 8455–8460, 10.1158/0008-5472.CAN-05-1162 (2005). 16166325

[b7] Li X. *et al.* Enhanced cytotoxicity and activation of ROS-dependent c-Jun NH2-terminal kinase and caspase-3 by low doses of tetrandrine-loaded nanoparticles in Lovo cells–a possible Trojan strategy against cancer. European journal of pharmaceutics and biopharmaceutics : official journal of Arbeitsgemeinschaft fur Pharmazeutische Verfahrenstechnik e.V 75, 334–340, 10.1016/j.ejpb.2010.04.016 (2010). 20438840

[b8] Xu H. *et al.* An efficient Trojan delivery of tetrandrine by poly(N-vinylpyrrolidone)-block-poly(epsilon-caprolactone) (PVP-b-PCL) nanoparticles shows enhanced apoptotic induction of lung cancer cells and inhibition of its migration and invasion. International journal of nanomedicine 9, 231–242, 10.2147/IJN.S55541(2014) . 24403829 PMC3883593

[b9] Wan J. *et al.* Synergistic antitumour activity of sorafenib in combination with tetrandrine is mediated by reactive oxygen species (ROS)/Akt signaling. British journal of cancer 109, 342–350, 10.1038/bjc.2013.334 (2013). 23807172 PMC3721403

[b10] Li X. *et al.* Paclitaxel/tetrandrine coloaded nanoparticles effectively promote the apoptosis of gastric cancer cells based on "oxidation therapy". Molecular pharmaceutics 9, 222–229, 10.1021/mp2002736 (2012). 22171565

[b11] Liebmann J., Cook J. A. & Mitchell J. B. Cremophor EL, solvent for paclitaxel, and toxicity. Lancet 342, 1428 (1993). 7901713 10.1016/0140-6736(93)92789-v

[b12] Negishi T. *et al.* NK105, a paclitaxel-incorporating micellar nanoparticle, is a more potent radiosensitising agent compared to free paclitaxel. British journal of cancer 95, 601–606, 10.1038/sj.bjc.6603311 (2006). 16909136 PMC2360685

[b13] Ren F. *et al.* Paclitaxel-loaded poly(n-butylcyanoacrylate) nanoparticle delivery system to overcome multidrug resistance in ovarian cancer. Pharmaceutical research 28, 897–906, 10.1007/s11095-010-0346-9 (2011). 21184150

[b14] Ding D. *et al.* Tumor accumulation, penetration, and antitumor response of cisplatin-loaded gelatin/poly(acrylic acid) nanoparticles. ACS applied materials & interfaces 4, 1838–1846, 10.1021/am300138z (2012). 22364315

[b15] Wang H. *et al.* Self-assembly-induced far-red/near-infrared fluorescence light-up for detecting and visualizing specific protein-Peptide interactions. ACS nano 8, 1475–1484, 10.1021/nn4054914 (2014). 24417359

[b16] Li X. L. *et al.* Enhanced *in vitro* and *in vivo* therapeutic efficacy of codrug-loaded nanoparticles against liver cancer. International journal of nanomedicine 7, 5183–5190, Doi 10.2147/Ijn.S34886 (2012). 23055730 PMC3464082

[b17] Emerich D. F. *et al.* Sustained release chemotherapeutic microspheres provide superior efficacy over systemic therapy and local bolus infusions. Pharmaceutical research 19, 1052–1060 (2002). 12180539 10.1023/a:1016434926649

[b18] Ding D. *et al.* Cisplatin-loaded gelatin-poly(acrylic acid) nanoparticles: synthesis, antitumor efficiency *in vivo* and penetration in tumors. European journal of pharmaceutics and biopharmaceutics: official journal of Arbeitsgemeinschaft fur Pharmazeutische Verfahrenstechnik e.V 79, 142–149, 10.1016/j.ejpb.2011.01.008 (2011). 21272637

[b19] Ding D. *et al.* Nanospheres-incorporated implantable hydrogel as a trans-tissue drug delivery system. ACS nano 5, 2520–2534, 10.1021/nn102138u (2011). 21428432

[b20] Sakamoto J., Matsui T. & Kodera Y. Paclitaxel chemotherapy for the treatment of gastric cancer. Gastric cancer: official journal of the International Gastric Cancer Association and the Japanese Gastric Cancer Association 12, 69–78, 10.1007/s10120-009-0505-z (2009). 19562460

[b21] Gupta G. P. & Massague J. Cancer metastasis: building a framework. Cell 127, 679–695, 10.1016/j.cell.2006.11.001 (2006). 17110329

[b22] Bergers G. & Hanahan D. Modes of resistance to anti-angiogenic therapy. Nature reviews. Cancer 8, 592–603, 10.1038/nrc2442 (2008). 18650835 PMC2874834

[b23] Koontongkaew S. The tumor microenvironment contribution to development, growth, invasion and metastasis of head and neck squamous cell carcinomas. Journal of Cancer 4, 66–83, 10.7150/jca.5112 (2013). 23386906 PMC3564248

[b24] Huang B. *et al.* Downregulation of the GnT-V gene inhibits metastasis and invasion of BGC823 gastric cancer cells. Oncology reports 29, 2392–2400, 10.3892/or.2013.2373 (2013). 23563846

[b25] Bhullar J. S. *et al.* A true orthotopic gastric cancer murine model using electrocoagulation. Journal of the American College of Surgeons 217, 64–70 discussion 70-61, 10.1016/j.jamcollsurg.2013.01.062 (2013). 23583619

[b26] Shan Y. S. *et al.* Establishment of an orthotopic transplantable gastric cancer animal model for studying the immunological effects of new cancer therapeutic modules. Molecular carcinogenesis 50, 739–750, 10.1002/mc.20668 (2011). 20737421

[b27] Alexandre J. *et al.* Accumulation of hydrogen peroxide is an early and crucial step for paclitaxel-induced cancer cell death both *in vitro* and *in vivo*. International journal of cancer. Journal international du cancer 119, 41–48, 10.1002/ijc.21685 (2006). 16450384

[b28] Blanco E. *et al.* Colocalized delivery of rapamycin and paclitaxel to tumors enhances synergistic targeting of the PI3K/Akt/mTOR pathway. Molecular therapy : the journal of the American Society of Gene Therapy 22, 1310–1319, 10.1038/mt.2014.27 (2014). 24569835 PMC4088997

[b29] Li D. *et al.* Sox2 is involved in paclitaxel resistance of the prostate cancer cell line PC-3 via the PI3K/Akt pathway. Molecular medicine reports 10, 3169–3176, 10.3892/mmr.2014.2630 (2014). 25310235

[b30] Mabuchi S. *et al.* Inhibition of phosphorylation of BAD and Raf-1 by Akt sensitizes human ovarian cancer cells to paclitaxel. The Journal of biological chemistry 277, 33490–33500, 10.1074/jbc.M204042200 (2002). 12087097

[b31] Kim S. H., Juhnn Y. S. & Song Y. S. Akt involvement in paclitaxel chemoresistance of human ovarian cancer cells. Annals of the New York Academy of Sciences 1095, 82–89, 10.1196/annals.1397.012 (2007). 17404021

[b32] Fidanboylu M. & Griffiths L. A. & Flatters, S. J. Global inhibition of reactive oxygen species (ROS) inhibits paclitaxel-induced painful peripheral neuropathy. PloS one 6, e25212, 10.1371/journal.pone.0025212 (2011). 21966458 PMC3180385

[b33] Yang J. C. *et al.* Selective targeting of breast cancer cells through ROS-mediated mechanisms potentiates the lethality of paclitaxel by a novel diterpene, gelomulide K. Free radical biology & medicine 51, 641–657, 10.1016/j.freeradbiomed.2011.05.012 (2011). 21641992

[b34] Li X. *et al.* Enhanced *in vitro* and *in vivo* therapeutic efficacy of codrug-loaded nanoparticles against liver cancer. International journal of nanomedicine 7, 5183–5190, 10.2147/IJN.S34886 (2012). 23055730 PMC3464082

