# Supplementary material for: Efficient antitumor effect of co-drug-loaded nanoparticles with gelatin hydrogel by local implantation
Source: Sci Rep. 2016 May 26;6:26546. doi: 10.1038/srep26546 (PMC4880919; doi:10.1038/srep26546)
Supplement: Supplementary Information [file srep26546-s1.doc]

**Supplement**

**Efficient antitumor effect of co-drug-loaded nanoparticles with gelatin hydrogel by local implantation**

*Hao Zhang ^1a^, Yong Tian ^2a^, Zhenshu Zhu ^3,4^, Huae Xu ^5^, Xiaolin Li ^1^*, Donghui Zheng ^6^*, Weihao Sun ^1*^*

^1^ Department of Geriatrics, the First Affiliated Hospital to Nanjing Medical University, Nanjing, 210029, China

^2^ Department of Orthopaedics, Jiangsu Taizhou People’s Hospital, Taizhou, 225300, China

^3^ Key Laboratory of Drug Quality Control and Pharmacovigilance (China Pharmaceutical University), Ministry of Education, Department of Pharmaceutical Analysis, China Pharmaceutical University, Nanjing 210009, China

^4^ Department of Chemical and Biomolecular Engineering, National University of Singapore, Singapore, 117585

^5^ Department of Pharmacy, the First Affiliated Hospital to Nanjing Medical University, Nanjing, 210029, China

^6^ Department of Nephrology, Huai'an Hospital Affiliated with Xuzhou Medical College and Huai'an Second Hospital, Huai'an 223002, China

^a^ These authors contributed equally to this article.

*Correspondence to: Xiaolin Li, Tel.: +86-25-68135151; Fax: +86-25-83780170; E-mail address: lxl@njmu.edu.cn or Donghui Zheng, Tel.: +86-517-83668282; Fax: +86-517-83668282, E-mail address: zdh2014@xzmc.edu.cn or Weihao Sun, Tel.: +86-25-68135153; Fax: +86-25-83780170; E-mail address: swh@njmu.edu.cn

**Method for Fig. 1S:** Mean diameter and size distribution of the drug loaded nanoparticles (P/T-NPs) before and after loaded into the gel were measured, respectively, by photon correlation spectroscopy (dynamic light scattering [DLS]) using a Brookhaven BI-9000 AT instrument (Brookhaven Instruments Corporation, Holtsville, NY, USA).


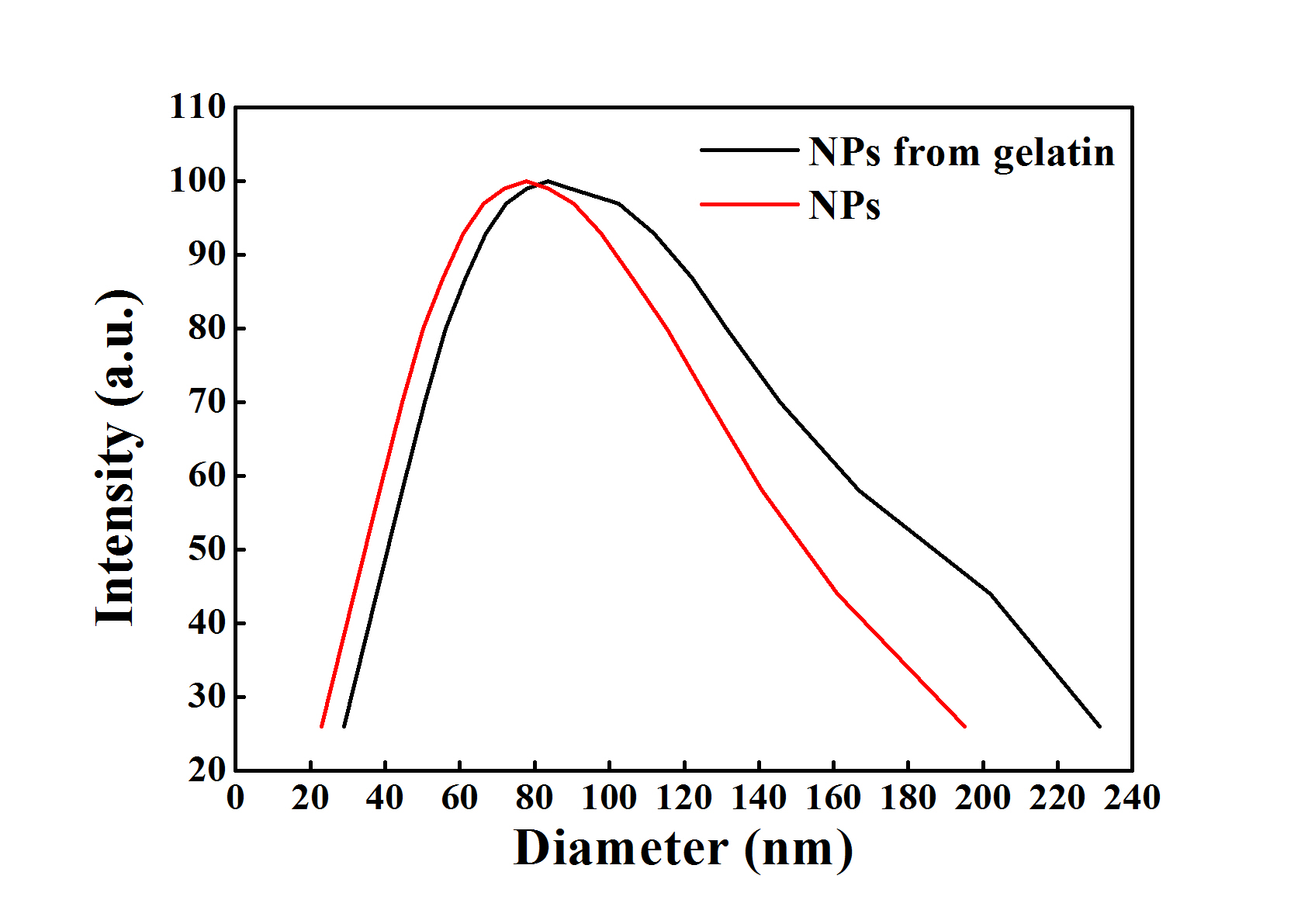


**Fig. 1S.** Particle size and distribution of NPs before and after loaded into the gels.

**Methods for Fig. 2S :** To further determine if there was any Tet or Ptx in gelatin instead of in the nanoparticles, ultra-high amount of gelatinase was prepared freshly and added into the gelatin gel containing drug loaded NPs. Fully degradation of the gel could be seen after only 15 mins. Then the drug loaded nanoparticles were harvested via centrifugation followed by the detection of the content of Ptx and Tet in the supernatant by HPLC as described in the part of 2.4.


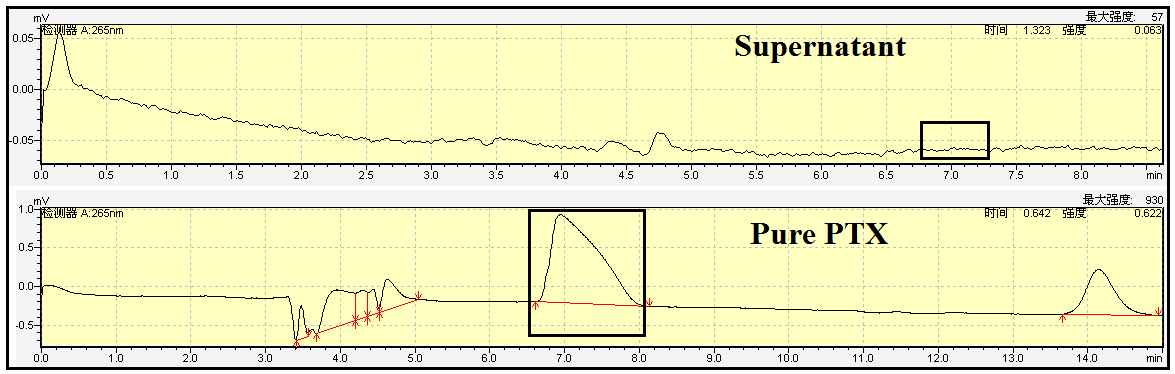


**Fig. 2S.** HPLC spectra of Ptx in the supernatant. The upper panel: HPLC spectra of the supernatant. The lower panel: HPLC spectra of pure Ptx.


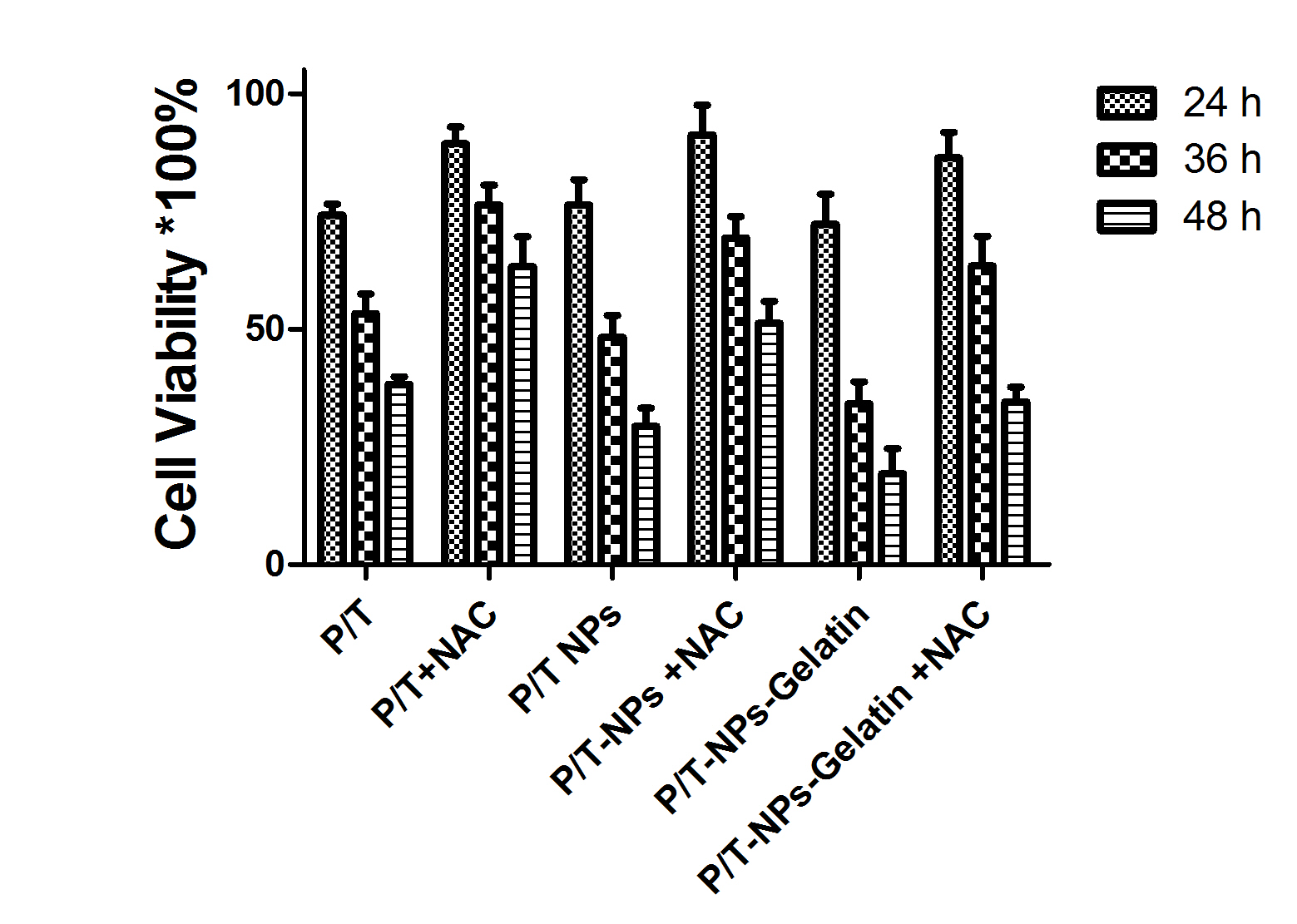


**Fig. 3S.** In vitro cytotoxicity of different agents on SGC-7901 cells.


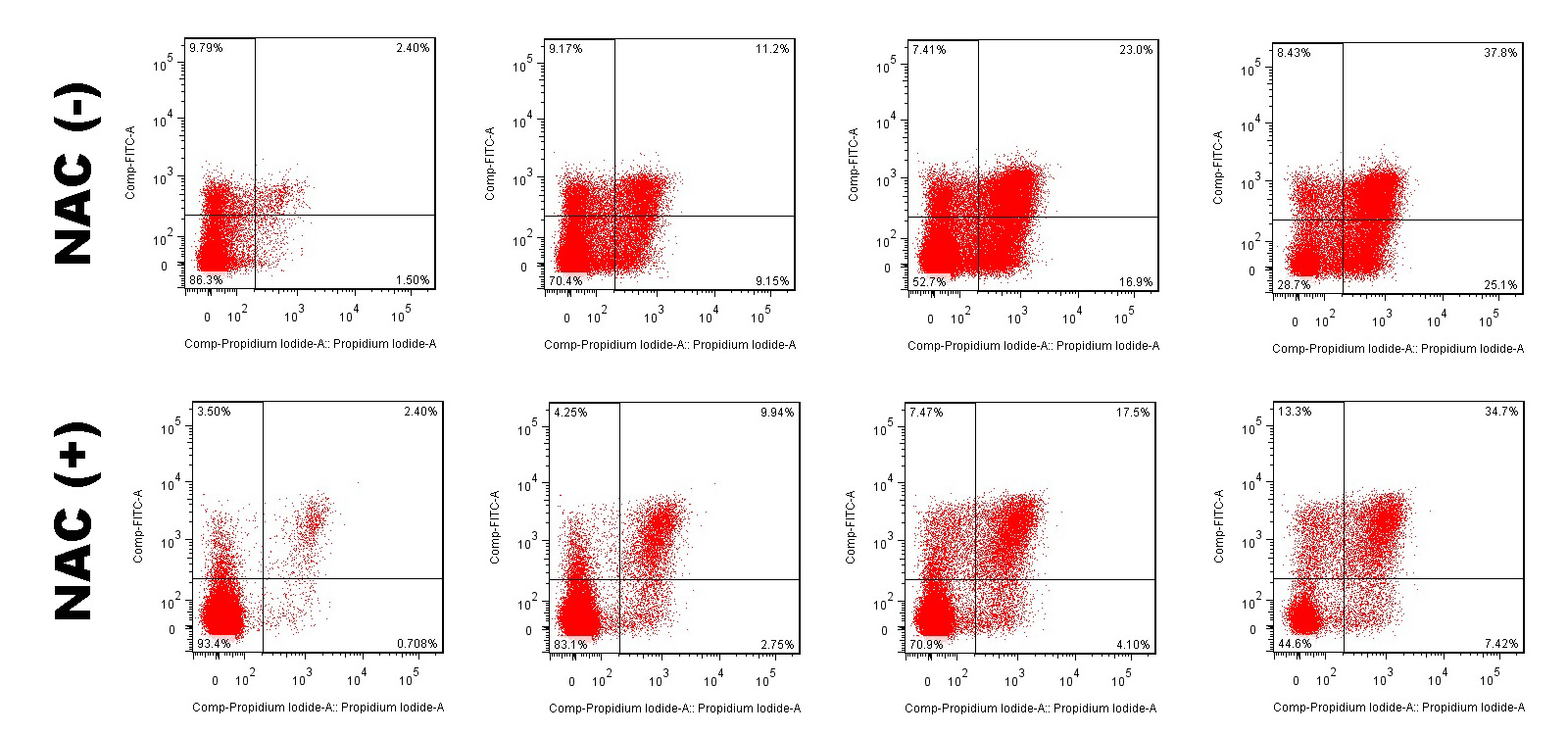


**Figure 4.** Induction of apoptosis by Ptx and Tet in different formulations for 48 h with or without the presence of NAC detected by FCAS.

**Tab. 1.** The values of IC50 of Ptx/Tet combination on BGC-823 cells for 48 h.

| **IC 50 (48 h)** | **P/T** |  |  |
| --- | --- | --- | --- |
| **Ptx (nM)** | 89.4±4.2 |  |  |
| **Tet (μM)** | 9.5±0.5 |  |  |

**Tab. 2.** The values of IC50 of Ptx/Tet combination on SGC-7901 cells for 48 h.

| **IC 50 (48 h)** | **P/T** |  |  |
| --- | --- | --- | --- |
| **Ptx (nM)** | 73.4±2.4 |  |  |
| **Tet (μM)** | 7.5±0.6 |  |  |
